# Hierarchical Area-Based and Path-Based Heuristic Approaches for Multirobot Coverage Path Planning with Performance Analysis in Surveillance Systems

**DOI:** 10.3390/s23208533

**Published:** 2023-10-17

**Authors:** Junghwan Gong, Seunghwan Lee

**Affiliations:** School of Electronic Engineering, Kumoh National Institute of Technology, Gumi 39177, Republic of Korea; 20236005@kumoh.ac.kr

**Keywords:** ant colony optimization, genetic algorithm, multirobot coverage path planning, traveling salesman problem

## Abstract

In this study, we present a systematic exploration of hierarchical designs for multirobot coverage path planning (MCPP) with a special focus on surveillance applications. Unlike conventional studies centered on cleaning tasks, our investigation delves into the realm of surveillance problems, specifically incorporating the sensing range (SR) factor equipped on the robots. Conventional path-based MCPP strategies considering SR, primarily rely on naive approaches, generating nodes (viewpoints) to be visited and a global path based on these nodes. Therefore, our study proposes a general MCPP framework for surveillance by dealing with path-based and area-based structures comprehensively. As the traveling salesman problem (TSP) solvers, our approach incorporates not the naive approach but renowned and powerful algorithms such as genetic algorithms (GAs), and ant colony optimization (ACO) to enhance the planning process. We devise six distinct methods within the proposed MCPP framework. Two methods adopt area-based approaches which segments areas via a hierarchical max-flow routing algorithm based on SR and the number of robots. TSP challenges within each area are tackled using a GA or ACO, and the result paths are allocated to individual robots. The remaining four methods are categorized by the path-based approaches with global–local structures such as GA-GA, GA-ACO, ACO-GA, and ACO-ACO. Unlike conventional methods which requires a global path, we further incorporate ACO- or GA-based local planning. This supplementary step at the local level enhances the quality of the path-planning results, particularly when dealing with a large number of nodes, by preventing any degradation in global path-planning outcomes. An extensive comparative analysis is conducted to evaluate the proposed framework based on execution time, total path length, and idle time. The area-based approaches tend to show a better execution time and overall path length performance compared to the path-based approaches. However, the path-based MCPP methods have the advantage of having a smaller idle time than the area-based MCPP methods. Our study finds that the proposed area-based MCPP method excels in path planning, while the proposed path-based MCPP method demonstrates superior coverage balance performance. By selecting an appropriate MCPP structure based on the specific application requirements, leveraging the strengths of both methodologies, efficient MCPP execution becomes attainable. Looking forward, our future work will focus on tailoring these MCPP structures to diverse real-world conditions, aiming to propose the most suitable approach for specific applications.

## 1. Introduction

Recently, research on multirobot coverage path planning (MCPP) has been actively conducted for various purposes such as unmanned large space surveillance, cleaning, lawn mowing, and crevasse exploration [1,2,3,4,5]. Among them, cleaning and lawn mowing are tasks that are only allowed to directly visit all areas, regardless of the sensing range (SR) of the robot. The SR is the detection range of the sensor mounted on the robot. If a LiDAR sensor is employed in outdoor environments, it usually has an SR of 30 m or more. It usually covers a given area in the form of a boustrophedon decomposition [6]. However, surveillance and exploration problems aim to monitor, explore, and verify the given areas perfectly based on the SR of the sensors mounted on the robots within a limited time. To deal with this problem, SR-based viewpoints and various MCPP methods are considered together.

Historically, MCPP originates from the path-planning (PP) problem. PP refers to the process of planning a safe path for a robot to move from one point to another without colliding with obstacles [7]. If the robot utilizes its onboard sensors to cover the entire given area, the viewpoint generation is based on SR, and PP can be performed to ensure that all viewpoints are visited. In this case, the problem of visiting all viewpoints with minimal cost is defined as the traveling salesman problem (TSP). Coverage path planning (CPP) encompasses this series of processes [8]. Technically, CPP deals with viewpoint generation for the solution of the TSP for a single robot. MCPP, as a form of CPP for multiple robots, is divided into a path-based approach and an area-based approach [9]. The path-based approach refers to dividing the results of CPP into *m* robot allocations evenly, which can be seen as a multiple traveling salesman problem (mTSP). The area-based approach involves dividing the entire area into *m* regions and performing single-robot CPP for each region. Each approach may have advantages and disadvantages in terms of path uniformity, computational effort, and path planning. However, no research has been conducted to include them in a surveillance system in detail. In this study, we comprehensively deal with both path-based and area-based approaches in the general MCPP framework for surveillance systems.

In the problem of MCPP, the visiting points (nodes) and the distance information between them (edges) are represented by a graph. Therefore, the core of CPP is finding the optimal path for the graph-represented area. However, most TSPs are NP-hard problems. Thus, heuristic methods are required to solve them [10]. There are many types of heuristic algorithms. In this study, we consider genetic algorithm (GA) [11] and ant colony optimization (ACO) [12] approaches, which are representative heuristic algorithms with good characteristics that solve optimization problems and can be easily combined with various techniques to solve the TSP of CPP. A GA demonstrates a rapid initial convergence. However, it slows down subsequently due to the absence of a positive feedback mechanism. Consequently, a GA excels in solving a limited number of TSP instances. On the other hand, ACO offers the advantage of achieving optimal results for large and intricate problems. However, it suffers from poor performance initially because of insufficient pheromone levels [13]. The positive feedback mechanism, represented by the accumulation of pheromones, accelerates the convergence speed, enabling ACO to handle complex tasks more efficiently [11,14]. Although these methods have mainly been utilized to solve single-robot CPP, this study presents four combinations of these two methods (GA-GA, GA-ACO, ACO-GA, ACO-ACO) for MCPP and compares their performance in terms of environment (edge shape) and the number of visiting points (node count) variations.

This study contributes in the following ways:

1. Design of hierarchical structures: We propose a hierarchical general MCPP framework for surveillance systems with area-based and path-based structures, integrating popular heuristic methods such as GAs and ACO. Unlike previous works, area-based structures are added in the general MCPP framework. In addition, more powerful heuristic methods are utilized instead of naïve approaches such as the nearest neighborhood approach.
2. Performance analysis: We conduct a comprehensive analysis of combination structures, considering factors like execution speed, total travel distance, and the sum of idle time. The last factor is related to the distribution of travel tasks among multiple robots.
3. In-depth method comparison: We provide a detailed comparison between area-based MCPP structure and path-based MCPP structure, evaluating their performance in terms of path planning and coverage balancing.

This study is structured as follows: Section 2 provides an overview of related MCPP research. In Section 3, GA and ACO methods and their capabilities in solving single-robot CPP problems are discussed. Section 4 presents the hierarchical combination structures for MCPP. Section 5 delves into comparative experiments under diverse environments, exploring the impact of changes in the number of robots and SR. Performance metrics include execution speed, total travel distance, and task distribution among robots. Section 6 analyzes each method’s path-planning and coverage-balancing performance. Finally, Section 7 concludes our study, offering insights into future research directions.

## 2. Related Work

Research on CPP is being conducted in various fields, including sanitation robots [15], harvesting robots [16], vacuum cleaning robots [17], and surveillance robots [3]. These studies primarily focus on coverage completeness and minimizing the overlap of coverage paths [18,19,20]. In CPP research, single robot CPP normally focuses on coverage completion, but MCPP focuses on a uniform task allocation among multiple robots, minimizing overall work time, energy efficiency, and so on. Among CPP studies, MCPP research presents a challenging problem due to the consideration and cooperation between multiple robots [21]. However, compared to single-robot CPP, MCPP offers advantages such as the parallel completion of tasks, a reduced time consumption, and improved efficiency. For example, if a single robot and multiple *m* robots perform CPP in the same environment, each robot visits only one node at the same rate, but *m* robots can visit *m* nodes at once. As the number *m* increases, the efficiency also increases. In addition, it is possible to plan for m−1 robots in the event of a breakdown or charging, but it is difficult to keep performing coverage in the case of a single robot.

The key focus of MCPP research lies in solving the mTSP problem. MCPP research can be categorized into path-based and area-based approaches [9]. For MCPP, the area-based method first divides the entire area for *m* robots and then proceeds with planning for each area. In contrast, the path-based method first generates a global path for all nodes to be visited (viewpoints) and then assigns each path by dividing the global path according to the number of robots, *m*. For area-partitioning, methods such as Lloyd’s algorithm [22], Voronoi partitioning [23], K-means [24], heuristic techniques [25], and the alternate-offer protocol [26] have been explored. Technically, Lloyd’s algorithm and Voronoi partitioning focus on region partitioning. In addition, they do not take SR into account. Since the K-means method is a method of dividing the area iteratively, it has the limitation of not being able to include the distribution of nodes considering SR. Ref. [25] dealt with the region segmentation of a polygonal map without considering both arbitrary maps and SR. Ref. [26] proposed a method to appropriately divide the region within a limited model. However, the study was not extended to maps with obstacles.

The DARP (Divide Areas based on Robots’ Initial Positions) method [27] assigns optimal regions to each robot based on the grid map and the initial positions of robots and obstacles. This method has been studied in combination with a spanning tree and ACO. However, while a preallocation of areas before path generation reduces computational complexity, it poses challenges in obtaining optimal solutions for MCPP.

Prominent studies in path-based approaches were introduced in [28,29], based on the art gallery problem (AGP) formulated by Klee [30]. The AGP involves placing a minimum number of necessary guards with sensing ranges to protect an art gallery. In these studies, the problem of placing guards was seen as a problem of generating necessary nodes (viewpoints) to visit. The authors assumed a polygonal obstacle and then divided the map based on the vertices of the obstacle. Within each area, nodes were generated at equal intervals considering the SR of the robot. The resulting nodes formed the basis for constructing graphs. For MCPP, a cyclic coverage method was proposed. The algorithm found the shortest tour on the graph similar to solving TSP. The tour was distributed to the robots. In [3], these studies were enhanced and expanded for MCPP. The nodes to be visited were generated based on the normal vector direction of the given map by considering the sensing range (SR). It means any maps were available except polygon-style ones. Additionally, processes such as path partitioning and recombination were added to achieve balanced paths. In this study, the graph construction method using the normal vectors of the map [3] is also employed. Unlike previous works, area-based structures as well as path-based structures are considered in our general MCPP framework for surveillance systems. ACO and GAs, which are more powerful heuristic methods, are combined into hierarchical structures such as ACO-ACO, ACO-GA, GA-GA, and GA-ACO instead of naïve approaches such as the nearest neighborhood approach utilized in [3]. Harrath et al. [31] proposed a hybrid methodology utilizing ACO, two-opt, and a GA. ACO was used as a solution for the TSP problem, while a two-opt algorithm and a GA were employed to enhance the solution. This method focused on solving the mTSP problem using powerful heuristic methods such as ACO and GAs but is different from the general MCPP solution for surveillance including a viewpoint generation using SR and obstacles in the map. However, that work is similar to the core of the path-based approach with the ACO-GA structure in our work. In this study, we design and analyze area-based and path-based approaches in the general MCPP framework by structuring them in various ways using heuristic methods such as ACO and GAs.

## 3. Coverage Path Planning

To solve CPP, it is first necessary to build a graph, *G*, for the entire area. The nodes of the graph, *N*, are iteratively generated by considering the SR in the normal vector direction from the occupied region of a given map [3]. An edge has a value if two nodes are connected along the free space of the map. It is calculated as the Euclidean distance. From the constructed graph, the problem is described as follows [32]:

(1) $$min\sum_{i=1}^{N}\sum_{j=1,j\neq i}^{N}E_{ij}C_{ij},$$

(2) $$s.t.\sum_{j,j\neq i}C_{i,j}=1,\;i=1,2,\ldots ,N,$$

(3) $$\sum_{i,i\neq j}C_{i,j}=1,\;j=1,2,\ldots ,N,$$

where $E_{ij}$ is the edge between the *i*th node and *j*th node. $C_{ij}$ denotes connectivity ($C_{ij}\in \left\{0,1\right\}$) for i≠j. Therefore, the CPP problem lies in finding $C_{ij}$ that satisfies (1) in the given graph.

### 3.1. GA-BasedCPP Approach

When the graph is constructed, GA-based CPP proceeds in the following way. Given the initial paths(chromosomes) $P_{1}$ and $P_{2}$, a crossover is performed where the crossover rate is $R_{cross}$. A crossover is a structure that evolves as parental chromosomes cross over to create new chromosomes. At this time, the direction of evolution is to decrease the cost of the path that is calculated as the sum of the graph edges. Furthermore, the GA addresses the local minimum problem by performing a mutation operation of $R_{mu}$ probability, which causes a portion of the factor information on the chromosome to change to a random value, resulting in a different solution. The number of chromosomes, denoted as $M_{ch}$, is predetermined and employed in parallel computations. In this study, these specific parameters were determined from the research conducted in [11].

### 3.2. ACO-Based CPP Approach

ACO-based CPP operates as follows. Initially, a certain number of ants, denoted as $N_{ant}$, randomly select nodes from the constructed graph based on the initialization rules. Each ant chooses the next node to visit using the state transition rule and undergoes an iterative process of exploration. During this process, the ants update the amount of pheromone on each visited node according to the local updating rule. After completing the exploration phase, when all ants have finished their traversal, the amount of pheromone is further updated based on the global updating rule. The amount of the update is determined by the following parameters: α (pheromone coefficient), β (heuristic coefficient), and ρ (pheromone evaporation rate). Larger values of α increase the dependency on the ant’s pheromone information when performing CPP, while larger values of β increase the dependency on heuristic calculations. For ρ, decreasing the value reduces the evaporation rate of the pheromone, making the information in the pheromone last longer as the generation evolves. Finally, each ant constructs a navigation path by considering both heuristic information to select nearby nodes and pheromone information to select nodes with higher amounts of pheromones. This entire process is repeated for a specified number of iterations.

The performance analysis of applying ACO to the TSP by varying the parameters is conducted in [12]. Ref. [12] analyzed ACO by changing parameters in order to obtain optimal ACO parameter values to solve various TSP problems. Based on this, this study utilized the resulting parameter values.

## 4. Multirobot Coverage Path Planning

**Figure 1:**
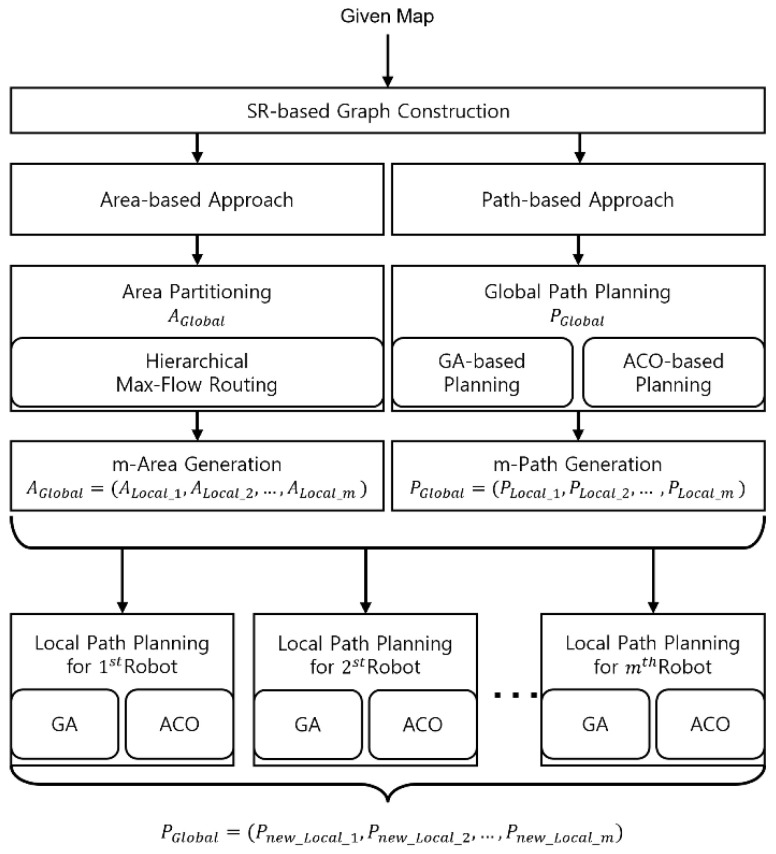
The flowchart of the proposed MCPP structures. This structure constructs an SR-based graph of information about a given map. Subsequently, the solution of the multiple TSPs shows how multirobot coverage performs with the chosen approach using the constructed graph.

Figure 1 illustrates the flowchart of the proposed MCPP structures. The entire coverage area, referred to as $A_{Global}$, is divided and allocated to *m* robots as individual task quotas ($A_{Global}$ = {$A_{Local\_1}$, $A_{Local\_2}$, …, $A_{Local\_m}$}. The process begins with the given map, from which an SR-based graph is constructed. Subsequently, the multiple TSPs are solved based on the chosen approaches.

In the area-based approach, the entire area is initially divided into partitions based on the number of robots, *m*. Each resulting partition is treated as a separate TSP, which is then solved using a heuristic method.

In the path-based approach, a global path is planned for all *N* viewpoints. This path is subsequently evenly divided into segments according to the value of *m*, and a replanning process using the heuristic method is performed for each segment.

The subsequent subsections provide a detailed explanation of each approach method.

### 4.1. Area-Based MCPP

In the area-based MCPP method, the entire coverage area, referred to as $A_{Global}$, is divided and allocated to *m* robots as individual task quotas ($A_{Global}$ = {$A_{Local\_1}$, $A_{Local\_2}$, …, $A_{Local\_m}$}). $A_{Local\_i}$ represents the subarea allocated to the *i*th robot after area partitioning processing. The partitioning of $A_{Global}$ is achieved using the hierarchical max-flow routing [33] method. The viewpoints that are encompassed within each area serve as the input for local path planning ($N\in A_{Local}$). Following several rounds of local path planning, each path is assigned to robots utilizing either a GA or ACO. Consequently, the resulting paths are denoted as $P_{Global}=\left\{P_{new\_Local\_1},P_{new\_Local\_2},\ldots ,P_{new\_Local\_m}\right\}$.

### 4.2. Path-Based MCPP

The path-based MCPP method involves conducting global coverage path planning using either a GA or ACO on a given graph. This is because the individual coverage path of each robot is obtained from a certain portion of the global path. The resulting global path denoted as $P_{Global}$ consisting of ordered nodes can be evenly divided into segments based on the specified number of robots, *m*, resulting in $P_{Global}=\left\{P_{Local\_1},P_{Local\_2},\ldots ,P_{Local\_m}\right\}$. For example, if $P_{Global}$ consists of 20 sorted nodes for 4 robots, then each $P_{Local}$ has 5 sorted nodes. Subsequently, a GA or ACO is applied once again to each divided path to obtain an optimized path for each segment, resulting in $P_{Global}=\left\{P_{new\_Local\_1},P_{new\_Local\_2},\ldots ,P_{new\_Local\_m}\right\}$. Thus, depending on the chosen methodologies for global path planning and local path planning, four possible structures can be organized: ACO-ACO, ACO-GA, GA-GA, and GA-ACO. These are all possible structures.

The complexity of the TSP is regarded as $O(N^{2}LogN)$ for *N* nodes. Hence, in scenarios with a large number of nodes, the MCPP approach with heuristic approaches for the complexity of TSP may not achieve optimality due to the persistence of local minimum problems. This can cause the MCPP approach to fail to achieve optimality. Nevertheless, in our path-based approach, after assigning a path to an individual robot, local planning is additionally performed for a small number of individual nodes. This increases the chance of finding a better solution that would not have been found during global path planning for all nodes. Consequently, the hierarchical structure proposed in this study can provide slightly improved outcomes for individual paths.

In the path-based approach, after assigning a path to an individual robot, local planning is performed once again for a small number of individual nodes. This increases the chance of finding a better solution that would not have been found during global path planning for all nodes.

## 5. Experiment

In this section, various experiments performed for the evaluation of the proposed MCPP structure are presented. The experiments were carried out in three environments: simple, partially complex, and complex environments. The proposed MCPP structures were evaluated depending on the number of nodes (*N*) and number of robots (*m*). Three evaluation factors were used in the evaluation: the average algorithm execution time, the total distance traveled by the robot, and the idle time of the robot.

Prior to the experiment, the aforementioned parameter values of the ACO and GA are presented in detail in Table 1. As for the values of the parameters, we found efficient parameter values by referring to the results of analyzing the effect of changing the parameters in [11,12]. $N_{ant}$ is the number of ants, *N* is the number of nodes, $R_{mu}$ is the probability of mutation, $R_{cross}$ is the probability of crossover, and $M_{ch}$ is the number of initial chromosomes.

In the experiments, a total of six methods were employed, comprising area-based methods consisting of either ACO or a GA, as well as path-based methods with four combinations of ACO and GAs. Furthermore, three different maps were tested, each varying in complexity. The performance of the six methods was evaluated by varying the number of robots and nodes based on each map. To assess performance, the path planning efficiency of each algorithm was compared using the average algorithm execution time, $T_{avg}$, and the total distance covered by the robots, $L_{sum}$. Additionally, the idle time of the robots was computed to represent the effectiveness of their utilization, denoted as $T_{idle}$. For both $L_{sum}$ and $T_{avg}$, the smaller the factors, the less distance the robot can travel and the less time it takes to compute. Thus, those factors show the coverage-path-planning performance. For $T_{idle}$, the smaller the value, the smaller the difference between the workloads (coverage nodes) of the robots, which shows the coverage-balancing performance. The formulas for calculating $L_{sum}$ and $T_{idle}$ are as follows:

(4) $$L_{sum}=\frac{1}{M}\sum_{k=1}^{M}\sum_{j=1}^{m}\sum_{i=1}^{N(k)-1}E_{P_{Local\{j,i\}},P_{Local\{j,i+1\}}}\;,$$

(5) $$T_{idle}=\frac{1}{V}\frac{1}{m}\sum_{i=1}^{m}\left(D_{max}-D_{i}\right),$$

where *M* is the total number of experiments, *m* is the number of robots, $P_{Local}$ is the waypoint of each robot, N(k) is the size of the generated $P_{Local}$ of the *k*th robot, $D_{i}$ is the path length of the *i*th robot, $D_{max}$ is the path length of the longest robot path among the robots’ paths, and *V* is the movement speed of the robot.

The environments utilized in the experiments were all 4704 × 3968 pixels in size. The experiments were performed using a robot with a movement speed of V = 1 pixel/s.

### 5.1. Simple Environment

**Figure 2:**
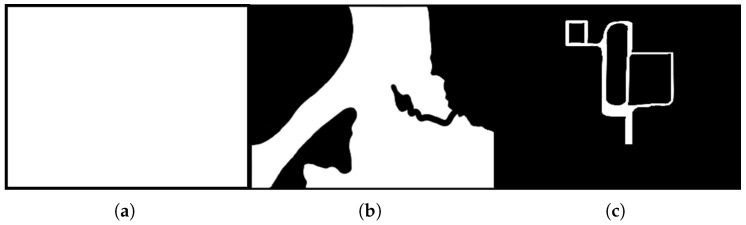
Three maps for the experiments. Each map differs in complexity. (**a**) Simple environment; (**b**) partially complex environment; (**c**) complex environment.

A simple environment is a simple structure with no obstacles, as shown in Figure 2a. When nodes are created in such an environment, there is a strong connectivity between them. It means that the robot can reach any node from the current node. Therefore, even if the optimal node selection fails at an arbitrary node, there is a high probability that the suboptimal selection can easily be found. Experimenting in this environment, we can infer that the global path planning performed only by the path-based MCPP method can result in a smaller $T_{avg}$ than the area-based MCPP method. In addition, if the number of robots increases or the number of nodes decreases, the $T_{avg}$ of the overall MCPP structure may decrease because the computational factor decreases.

**Figure 3:**
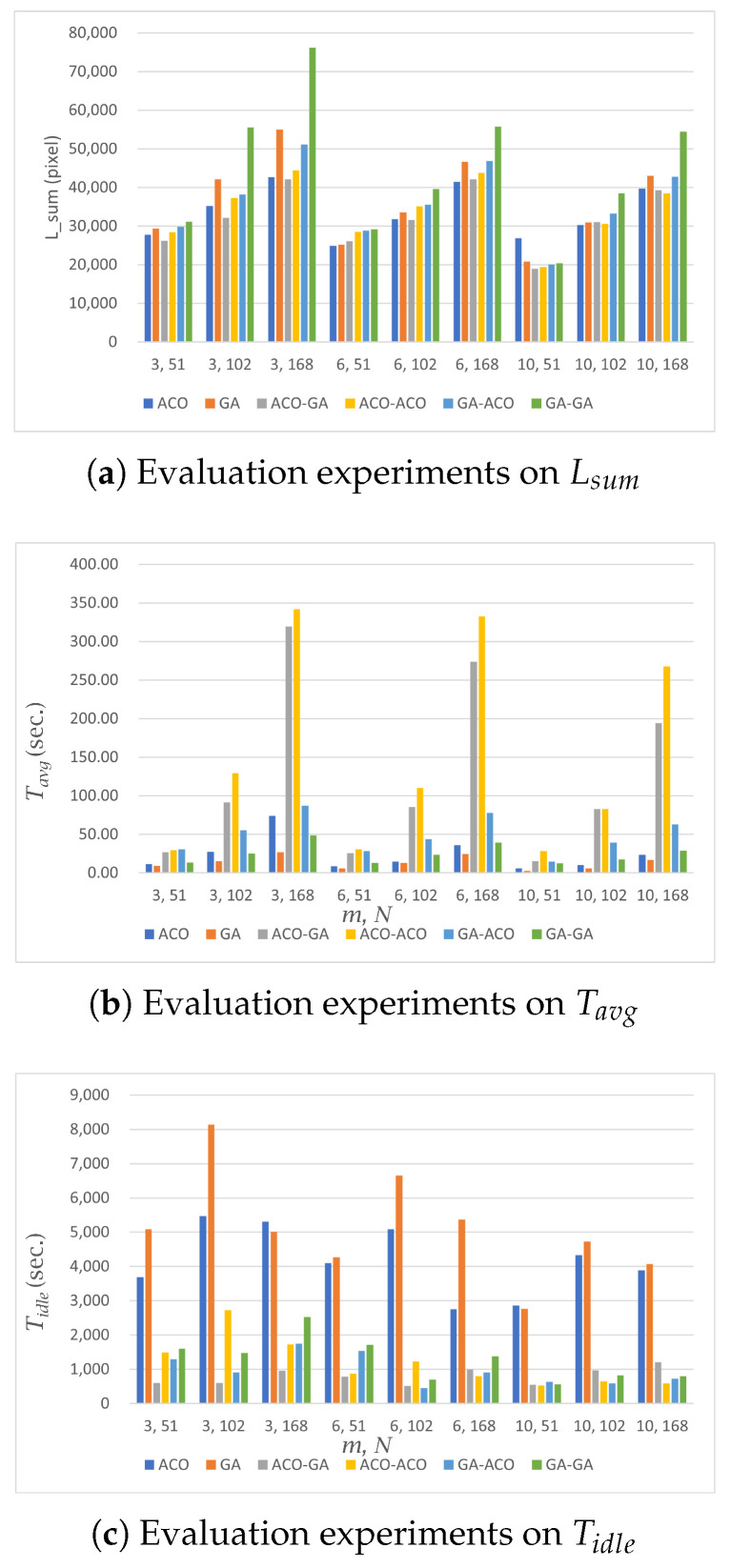
Performance evaluation in a simple environment. Each algorithm was evaluated in terms of $L_{sum}$, $T_{avg}$, and $T_{idle}$. The horizontal axis in each figure means (*m*, *N*), and the vertical axis denotes the measured distance or time according to the evaluation criteria.

The results of our experiments in the simple environment are shown in Table 2 and Figure 3.

#### 5.1.1. Experimental Results: Node and Robot Variations (*N* and *m*)

For all algorithms performed in the simple environment, $T_{avg}$ and $L_{sum}$ increased as the number of nodes increased. This is because as the number of nodes increases, the number of viewpoints the robot must pass through increases. In addition, the number of possible paths that can be generated increases, resulting in an increase in $T_{avg}$ and $L_{sum}$.

In both the area-based and path-based MCPP methods, $L_{sum}$ and $T_{avg}$ tended to decrease as the number of robots increased. This outcome can be attributed to the fact that with a higher number of robots, the nodes are distributed among more robots, resulting in a reduced number of nodes assigned to each individual robot. Consequently, as the number of nodes assigned to each robot decreases, the overall coverage task becomes more efficient, leading to a decrease in both $L_{sum}$ and $T_{avg}$. The specific numerical results reflecting this trend can be observed in Table 2 and Figure 3.

#### 5.1.2. MCPP Methods

In the area-based MCPP method, the GA showed a $T_{avg}$ up to 37.81 s faster than the ACO, but for $L_{sum}$, the ACO showed an $L_{sum}$ of up to 5582 pixels shorter than the GA. The reason for this stems from the features of each algorithm, with the GA’s fast performance and ACO’s relatively better distance optimization performance than the GA.

In path-based MCPP, the path distance ($L_{sum}$) differences between the ACO-GA and ACO-ACO structures were similar overall. These two algorithms had relatively good $L_{sum}$ performance among all path-based MCPP methods. In terms of the performance for $T_{avg}$, the ACO-GA structure was up to 92.49 s faster than the ACO-ACO structure. As a result, the ACO-GA structure showed the best path-planning performance among path-based MCPP methods. For instance, the GA-GA method had the fastest execution time compared to other path-based MCPP methods, but its $L_{sum}$ was up to 26,467 pixels larger than that of the ACO-GA method, showing relatively worse performance among the path-based MCPP methods.

To compare the performance between the area-based MCPP approach and the path-based MCPP approach themselves, the area-based MCPP method with ACO and the path-based MCPP method with an ACO-GA structure, as their representatives, were considered. When considering $L_{sum}$, the results indicated that the path-based MCPP method achieved a shorter distance by up to 4461 pixels when the number of nodes, *N*, was 71. However, in experiments involving 104 and 162 nodes, the area-based MCPP method demonstrated a shorter distance by up to 1036 pixels. In the case of the path-based MCPP method, global path planning must be performed initially. When the number of nodes exceeded 100 in our test scenario, this method with ACO could not reach a fine solution as the computational complexity increased. This is due to a structural property of heuristic algorithms. In the case of the area-based MCPP method, even when there were more than 100 nodes generated, the coverage area was divided among the robots at first. Subsequently, the heuristic algorithm performed well for a relatively small number of nodes. This process led to a lower $L_{sum}$ compared to the path-based MCPP method. Regarding $T_{avg}$, the area-based MCPP method generally outperformed the path-based MCPP method with a noticeable performance difference of up to 198.61 s. This result represents that the area division process normally exhibits faster computation times than the global path-planning process according to increases in the number of nodes.

In terms of $T_{idle}$, the comparison results showed that the path-based MCPP method had a lower idle time compared to the area-based MCPP method with a difference of up to 2538 s. This implies that the path-based method more effectively deals with the given robots by minimizing their idle time.

These results comprehensively suggest that the area-based MCPP method provides advantages in terms of $L_{sum}$ and $T_{avg}$ while the path-based MCPP method excels in minimizing $T_{idle}$ and ensuring an efficient robot utilization.

#### 5.1.3. Graphical Results

**Figure 4:**
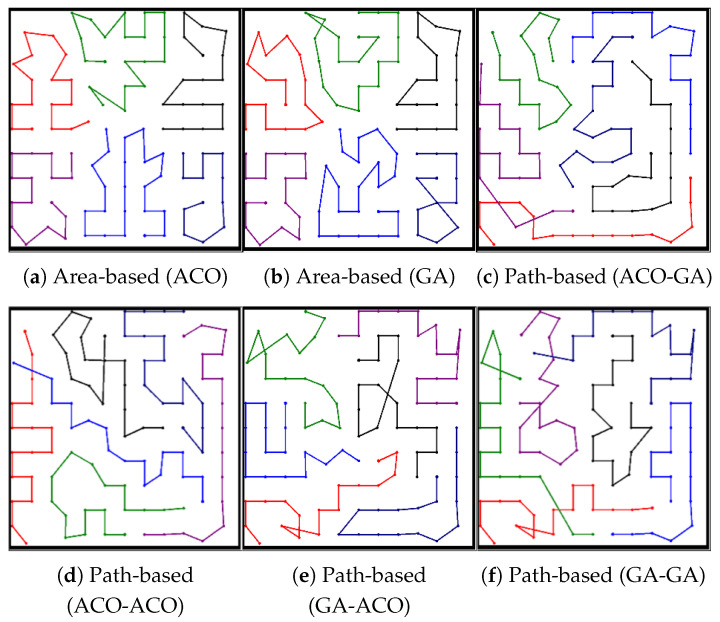
Experiment result in a simple environment. The results presented in (**a**–**f**) were obtained from experiments conducted using 102 nodes and 6 robots.

Figure 4 shows the representative results of coverage paths generated by each algorithm with *m* = 6 and *N* = 102. In the area-based MCPP method as shown in Figure 4a,b, the path on the bottom right is shorter than the paths of the other robots. For the path-based MCPP method depicted in Figure 4c–f, all robots are assigned paths consisting of the same number of nodes (*N* = 16), which results in a more efficient coverage-balancing performance compared to the area-based MCPP method. This implies that the area-based MCPP method exhibits a larger $T_{idle}$ compared to the path-based MCPP method, as previously discussed in the analysis of the results presented in Table 2 and Figure 3.

### 5.2. Partially Complex Environment

**Figure 5:**
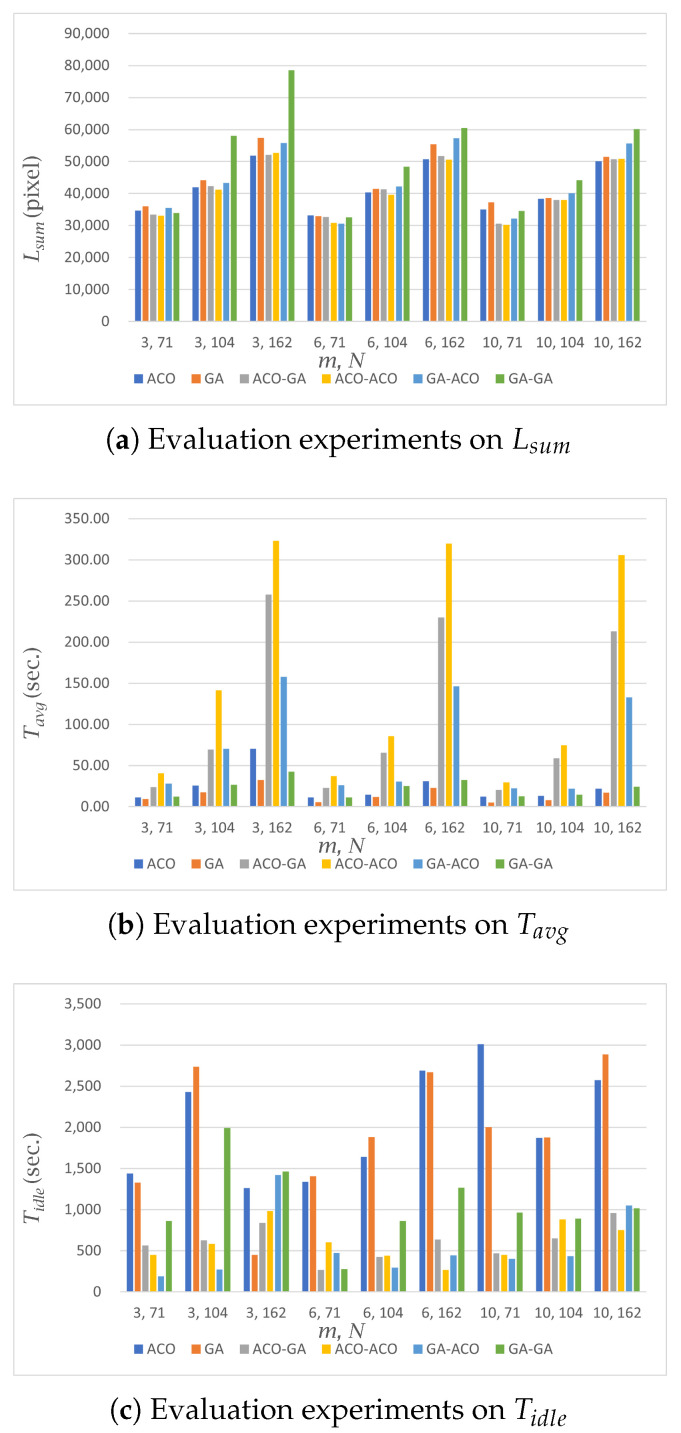
Performance evaluation in a partially complex environment. Each algorithm was evaluated in terms of $L_{sum}$, $T_{avg}$, and $T_{idle}$. The horizontal axis in each figure means (*m*, *N*), and the vertical axis denotes the measured distance or time according to the evaluation criteria.

A partially complex environment refers to a scenario where the entire environment is obstructed by certain terrain, as depicted in Figure 2b. In this case, the robot performing the coverage is limited to moving within the white area, while the black area represents regions where the robot is unable to navigate. When constructing graphs between nodes that are affected by these restricted areas, additional computational steps are required to account for the separation of such areas. Additionally, the connectivity between nodes tends to be weaker compared to a simple environment due to the introduction of restricted paths, which restrict the ability to find the next node from any given node. Table 3 and Figure 5 show the results of our experiments in a partially complex environment. The experimental results in the partially complex environment introduce an additional factor for calculating MCPP since there are more limited nodes compared to the simple environment. Therefore, the experimental results show that the performance difference in $L_{sum}$ between the GA and ACO increases more than in the simple environment.

#### 5.2.1. Experimental Results: Node and Robot Variations (*N* and *m*)

The results of the experiments with the number of nodes and robots in the partially complex environment were similar to those in the simple environment. It was observed that $L_{sum}$ and $T_{avg}$ changed proportionally with the number of nodes.

#### 5.2.2. MCPP Methods

In the area-based MCPP method, the performance difference in $T_{avg}$ between the GA and ACO was up to 47.31 s, which was 9.5 s higher than the time difference observed in the simple environment (37.81 s). Moreover, the performance difference in $L_{sum}$ was up to 12,340 pixels. This indicates that the performance difference more than doubled compared to the $L_{sum}$ difference observed in the simple environment (5582 pixels).

For the path-based MCPP method, we observed that the ACO-GA structure had the best distance performance with the smallest $L_{sum}$, and the GA-GA structure had the best time performance with the fastest $T_{avg}$. However, the GA-GA structure had an $L_{sum}$ of up to 34,061 pixels longer than the ACO-GA structure, indicating a worse path length optimization performance.

Comparing the ACO structure with the shortest $L_{sum}$ in the area-based MCPP method and the ACO-GA structure with the shortest $L_{sum}$ in the path-based MCPP method, it was found that $T_{avg}$ took up to 245.73 s longer in the path-based MCPP method compared to the area-based MCPP method. It showed that area-based MCPP method was relatively faster than the path-based one in computation time when performing path planning. On the other hand, $T_{idle}$ took up to 4867 s less in the path-based MCPP method compared to the area-based MCPP method. As in the simple environment, the path-based MCPP method had better coverage-balancing performance than the area-based MCPP method. Additionally, the area-based MCPP method exhibited $T_{idle}$ values ranging from 2749 to 5473 s, which was an increase from the $T_{idle}$ values observed in the simple environment (ranging from 1264 to 3009 s).

#### 5.2.3. Graphical Results

**Figure 6:**
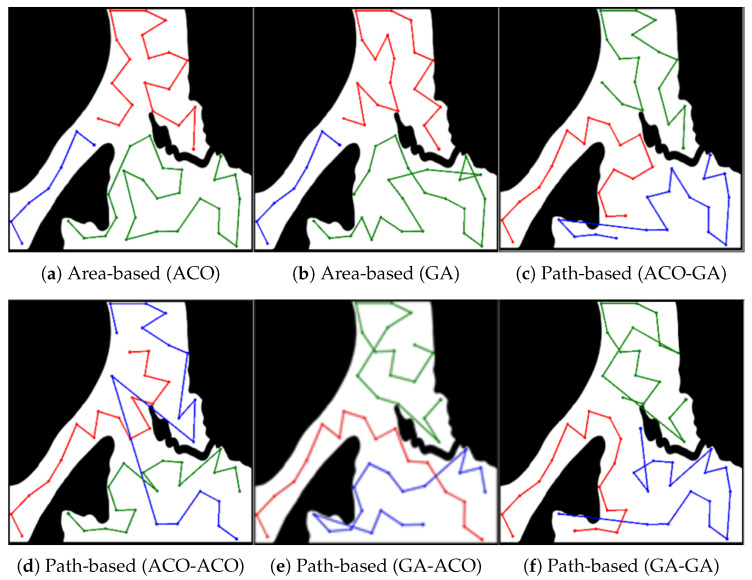
Experiment result in a partially complex environment. The results presented in (**a**–**f**) were obtained from the experiment conducted using 51 nodes and 3 robots.

The paths for each algorithm are depicted in Figure 6 with *m* = 3 and *N* = 51. In the case of the area-based MCPP method as shown in Figure 6a,b, the result of the paths on the bottom right reveals that Figure 6a exhibits a shorter path compared to Figure 6b. On the other hand, for the path-based MCPP method depicted in Figure 6c–f, the ACO-GA structure demonstrates the shortest $L_{sum}$ without any overlapping paths among different robots.

Among the path-based MCPP methods, except for the ACO-GA-structured algorithm, Figure 6d–f are not suitable for multirobot path planning, because there is a situation where different robots represent overlapping paths, which may cause collisions between robots. When comparing the different MCPP methods, it is observed that the path-based MCPP method achieved a more balanced and even distribution of paths compared to the area-based MCPP method.

### 5.3. Complex Environment

For the experiments conducted in a complex environment, we utilized an environment characterized by branching terrains where only white areas were accessible for movement, as illustrated in Figure 2c.

In this particular environment, the constructed graphs exhibited the most constrained edges between nodes compared to the previous experiments. Consequently, this environment displayed the weakest connectivity between nodes among all the experimental environments. The results of our experiments in the complex environment are shown in Table 4. In this environment, the MCPP optimization performance of the GA was significantly degraded due to the limited number of edges between most nodes, resulting in worse $L_{sum}$ values compared to ACO.

#### 5.3.1. Experimental Results: Node and Robot Variations (*N* and *m*)

**Figure 7:**
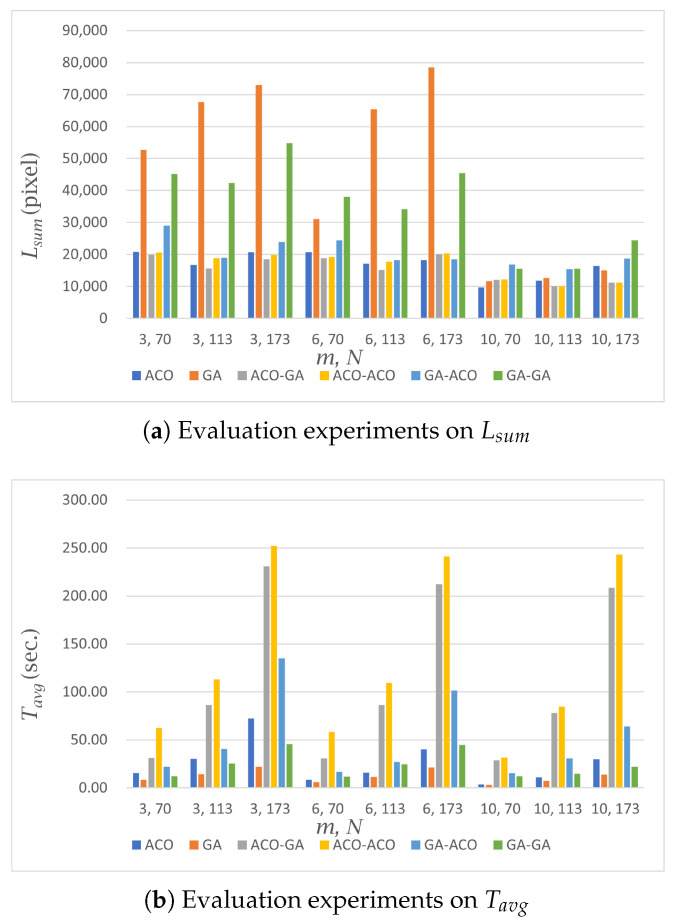

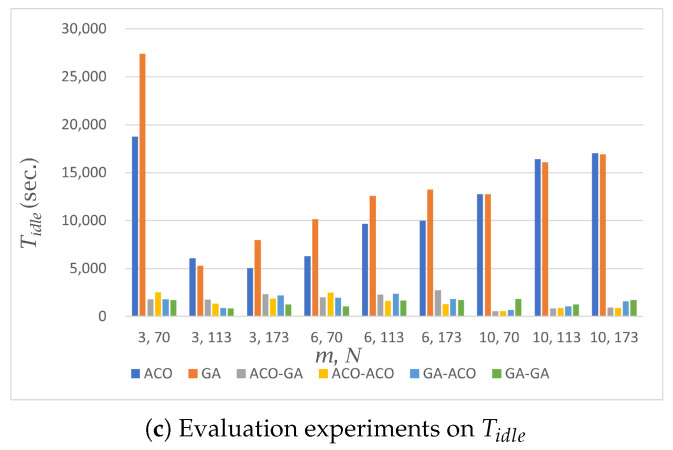
Performance evaluation in a complex environment. Each algorithm was evaluated in terms of $L_{sum}$, $T_{avg}$, and $T_{idle}$. The horizontal axis in each figure means (*m*, *N*) and the vertical axis denotes the measured distance or time according to the evaluation criteria.

Previous experiments performed in simple and partially complex environments generally showed that $L_{sum}$ increased as the number of nodes increased. However, Table 4 and Figure 7 show that for the area-based MCPP method with ACO and the path-based MCPP method, $L_{sum}$ became shorter as the number of nodes increased from 70 to 113. In the previous experiment, since there were many possible paths between the nodes, we were able to construct a sufficient number of edges to pass through all the nodes, resulting in a near-optimal $L_{sum}$. Therefore, as the number of nodes increased, the $L_{sum}$ also increased because there was no factor other than the number of nodes that affected the computation of the algorithm.

On the other hand, in the complex environment, the possible paths between the nodes were mostly constrained by obstacles, except for a few instances. When fewer than 100 nodes were created, there were situations where nodes were not assigned to branching points of the environment and instances where edges enabling traversal through all nodes were not constructed. It led to a poor distance optimization performance and a larger $L_{sum}$. However, as the number of nodes increased, the number of cases featuring passable paths between nodes increased, ensuring that all nodes had edges facilitating traversal. This improvement in distance optimization performance resulted in a reduced $L_{sum}$. In the other cases of this observation, the increase from 113 to 173 nodes yielded the same increase in $T_{avg}$ and $L_{sum}$ as in the previous experimental environments.

In the case of the number of robots, the results of the experiments in the complex environment were consistent with the previous experiments, with $L_{sum}$ and $T_{avg}$ decreasing as the number of robots increased.

#### 5.3.2. MCPP Methods

The area-based MCPP method using ACO exhibited a similar pattern to the previous experimental environments, with the ACO achieving a shorter $L_{sum}$ but longer $T_{avg}$ compared to the GA.

In the path-based MCPP methods, each algorithm structure resembled the experimental results in the partially complex environment, the ACO-GA structure demonstrating the lowest $L_{sum}$ and the GA-GA structure exhibiting the shortest $T_{avg}$. Analyzing the results from all experimental environments collectively, the average difference in $L_{sum}$ between the GA-GA and ACO-GA structures increased as the number of paths restricted by obstacles in the environment increased. In the simple environment, $L_{sum}$ was 26,467 pixels. In the partially complex environment, $L_{sum}$ was 36,061 pixels. Lastly, in the complex environment, $L_{sum}$ was 36,320 pixels.

When comparing the area-based MCPP method using ACO and the path-based MCPP method based on the ACO-GA structure, the path-based MCPP method displayed longer $T_{avg}$ and shorter $T_{idle}$ values compared to the area-based MCPP method, which aligned with the previous experimental results.

#### 5.3.3. Graphical Results

**Figure 8:**
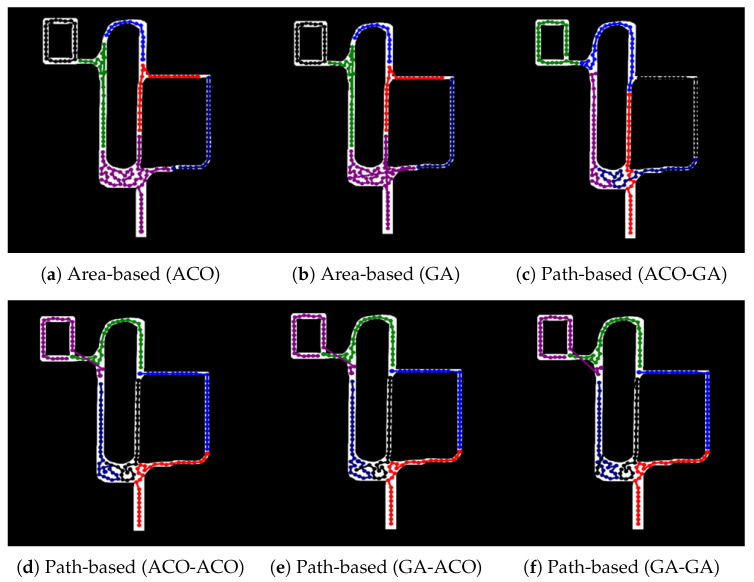
Experiment result in a complex environment. The results presented in (**a**–**f**) were obtained from the experiment conducted using 173 nodes and 6 robots.

Figure 8 shows the path results for each algorithm with *m* = 6 and *N* = 173. In Figure 8a,b, which represent the experimental results of the area-based MCPP method, the blue path at the top center is relatively shorter than the other paths. Based on this observation, the path-based MCPP method, depicted from Figure 8c–f, divides the paths more evenly compared to the area-based MCPP method, as shown in Figure 8a,b. Consequently, the path-based MCPP method yields smaller $T_{idle}$ values compared to the area-based MCPP method.

## 6. Discussion

To analyze the path-planning performance of MCPP, the results of $L_{sum}$ regarding two representative approaches were compared in experimental environments. As representative methods with good performance, a path-based MCPP method using ACO and an area-based MCPP method using an ACO-GA structure were chosen. The difference in $L_{sum}$ ranged from 257 pixels to 7907 pixels. When the number of nodes was more than 150, the experimental results showed that the area-based MCPP method had a smaller $L_{sum}$ than the path-based MCPP method regardless of the environmental complexity.

For $T_{avg}$, the difference in $T_{avg}$ performance between MCPP methods increased dramatically as the number of nodes increased. In other words, the area-based MCPP method showed relatively low $T_{avg}$ values compared to the path-based MCPP method. Unlike the path-based MCPP method, the area-based MCPP method did not require global CPP for all nodes. In the case of the area-based MCPP method, the area was divided quickly by the number of robots in advance, and then, a relatively small number of nodes was left in the divided area. Because the number of nodes was small, the heuristic algorithm achieved an optimal or near-optimal solution. This process improved both the computation time ($T_{avg}$) and the multirobot path-planning performance ($L_{sum}$).

To evaluate the performance of MCPP in terms of the efficiency of coverage-balancing performance, $T_{idle}$ for the representative methods were compared in the same manner as before. In all environments, the path-based MCPP method had a smaller $T_{idle}$ than the area-based MCPP method. In the simple environment, the difference in $T_{idle}$ ranged from 423 to 2538 s, with an average of 1424 s. In the partially complex environment, the difference in $T_{idle}$ ranged from 1756 to 4867 s, with an average of 3366 s. Lastly, in the complex environment, the difference in $T_{idle}$ ranged from 2745 to 16,951 s, with an average of 9647 s.

Considering these results comprehensively, the performance difference between the methods increases according to the increase in the environmental complexity. This is because path-based MCPP methods divide the path generated by the global path-planning process according to the number of robots. It increases the possibility that all robots will share the same amount of workload. On the other hand, the area-based MCPP method can divide the space irregularly depending on the type of environment. It means that as the environment becomes more complex, the nodes in the space are not divided evenly, and the coverage balancing efficiency deteriorates.

Through the above comprehensive analysis, the area-based MCPP approach shows its powerful performance in path planning whereas the path-based MCPP approach outperforms in coverage balancing.

## 7. Conclusions

In this study, we presented an analytical investigation of general MCPP structures for surveillance systems: area-based MCPP approaches and path-based MCPP approaches. To achieve this, we carefully designed hierarchical structures based on six different methods using the combination of ACO and GAs according to the approaches. In addition, we conducted experiments in various environments and several conditions. For performance evaluations, we introduced three evaluation metrics: $L_{sum}$, $T_{avg}$, and $T_{idle}$. These metrics allowed us to analyze the path-planning performance and coverage-balancing performance of each structure efficiently. As a result, the area-based MCPP structure showed its powerful performance in path planning, whereas the path-based MCPP approach outperformed in coverage balancing. Our future work will focus on applying these MCPP structures to a variety of real-world conditions with the goal of proposing the most suitable approach for specific applications. As a specific application, a number of robot operation tasks are currently being performed at Jang Bogo, South Korea’s high-tech Antarctic station.

## Figures and Tables

**Table 1:** Major parameters values for the experiments.

Algorithm	Parameter	Experimental Value
ACO	α	5
	β	1
	ρ	0.5
	$N_{ant}$	*N*
GA	$R_{mu}$	0.3
	$R_{cross}$	1
	$M_{ch}$	50

**Table 2:** Performance evaluation in a simple environment.

		**Area-Based MCPP Method (ACO)**			**Area-Based MCPP Method (GA)**			**Path-Based MCPP Method (ACO-GA)**		
***m***	***N***	$L_{\mathrm{sum}}\left(\mathrm{pixel}\right)$	$T_{\mathrm{avg}}\left(s\right)$	$T_{\mathrm{idle}}\left(s\right)$	$L_{\mathrm{sum}}\left(\mathrm{pixel}\right)$	$T_{\mathrm{avg}}\left(s\right)$	$T_{\mathrm{idle}}\left(s\right)$	$L_{\mathrm{sum}}\left(\mathrm{pixel}\right)$	$T_{\mathrm{avg}}\left(s\right)$	$T_{\mathrm{idle}}\left(s\right)$
3	71	34,679	11.23	1440	36,058	9.58	1330	33,435	23.77	567
	104	41,938	25.69	2428	44,145	17.46	2736	42,370	69.54	626
	162	51,854	70.33	1264	57,436	32.52	451	52,129	257.97	841
6	71	33,173	11.16	1339	32,953	5.38	1405	32,726	22.95	267
	104	40,353	14.72	1643	41,411	11.90	1881	41,336	65.60	428
	162	50,708	31.21	2691	55,470	22.80	2670	51,744	229.82	635
10	71	35,049	12.40	3009	37,225	5.04	2002	30,587	20.52	470
	104	38,420	13.14	1872	38,664	8.19	1875	37,955	58.94	650
	162	50,089	22.01	2573	51,434	17.23	2887	50,741	213.18	959
		**Path-Based MCPP Method (ACO-ACO)**			**Path-Based MCPP Method (GA-ACO)**			**Path-Based MCPP Method (GA-GA)**		
***m***	***N***	$L_{\mathrm{sum}}\left(\mathrm{pixel}\right)$	$T_{\mathrm{avg}}\left(s\right)$	$T_{\mathrm{idle}}\left(s\right)$	$L_{\mathrm{sum}}\left(\mathrm{pixel}\right)$	$T_{\mathrm{avg}}\left(s\right)$	$T_{\mathrm{idle}}\left(s\right)$	$L_{\mathrm{sum}}\left(\mathrm{pixel}\right)$	$T_{\mathrm{avg}}\left(s\right)$	$T_{\mathrm{idle}}\left(s\right)$
3	71	33,097	40.86	452	35,558	28.32	192	33,914	12.17	864
	104	41,246	141.47	584	43,289	70.28	272	58,020	26.77	1994
	162	52,709	323.38	983	55,743	157.69	1422	78,596	42.43	1465
6	71	30,879	37.38	605	30,604	26.34	476	32,557	11.59	278
	104	39,608	85.74	438	42,264	30.63	298	48,377	25.48	861
	162	50,623	319.65	268	57,284	146.50	443	60,517	32.66	1267
10	71	30,245	29.59	448	32,149	22.25	404	34,557	12.73	964
	104	37,983	74.76	880	40,092	22.01	434	44,128	14.73	892
	162	50,829	305.67	751	55,626	132.81	1052	60,152	24.44	1019

**Table 3:** Performance evaluation in a partially complex environment.

		**Area-Based MCPP Method (ACO)**			**Area-Based MCPP Method (GA)**			**Path-Based MCPP Method (ACO-GA)**		
***m***	***N***	$L_{\mathrm{sum}}\left(\mathrm{pixel}\right)$	$T_{\mathrm{avg}}\left(s\right)$	$T_{\mathrm{idle}}\left(s\right)$	$L_{\mathrm{sum}}\left(\mathrm{pixel}\right)$	$T_{\mathrm{avg}}\left(s\right)$	$T_{\mathrm{idle}}\left(s\right)$	$L_{\mathrm{sum}}\left(\mathrm{pixel}\right)$	$T_{\mathrm{avg}}\left(s\right)$	$T_{\mathrm{idle}}\left(s\right)$
3	51	27,711	11.35	3692	29,345	8.86	5088	26,218	26.72	603
	102	35,227	27.09	5473	42,147	15.02	8143	32,189	91.49	605
	168	42,655	73.81	5307	54,995	26.50	5018	42,107	319.54	959
6	51	24,870	8.78	4099	25,265	5.98	4276	26,146	25.63	792
	102	31,808	14.58	5092	33,620	12.91	6652	31,551	85.28	519
	168	41,492	36.23	2749	46,637	24.65	5374	42,080	274.05	993
10	51	26,899	6.07	2864	20,880	2.58	2763	18,992	14.90	556
	102	30,228	10.15	4329	30,977	5.87	4733	31,023	82.98	965
	168	39,699	23.29	3885	42,958	16.61	4075	39,314	193.95	1203
		**Path-Based MCPP Method (ACO-ACO)**			**Path-Based MCPP Method (GA-ACO)**			**Path-Based MCPP Method (GA-GA)**		
***m***	***N***	$L_{\mathrm{sum}}\left(\mathrm{pixel}\right)$	$T_{\mathrm{avg}}\left(s\right)$	$T_{\mathrm{idle}}\left(s\right)$	$L_{\mathrm{sum}}\left(\mathrm{pixel}\right)$	$T_{\mathrm{avg}}\left(s\right)$	$T_{\mathrm{idle}}\left(s\right)$	$L_{\mathrm{sum}}\left(\mathrm{pixel}\right)$	$T_{\mathrm{avg}}\left(s\right)$	$T_{\mathrm{idle}}\left(s\right)$
3	51	28,420	29.24	1494	29,885	30.76	1287	31,098	13.27	1602
	102	37,248	129.41	2729	38,135	55.32	904	55,505	24.87	1484
	168	44,428	342.09	1720	51,173	86.98	1745	76,168	48.48	2532
6	51	28,516	30.63	871	28,866	28.24	1534	29,176	12.78	1714
	102	35,125	110.43	1236	35,547	43.64	452	39,616	23.61	699
	168	43,833	332.39	802	46,838	77.69	907	55,793	39.12	1377
10	51	19,453	28.15	528	20,055	14.46	634	20,357	12.50	564
	102	30,579	82.86	646	33,257	39.27	584	38,513	17.54	822
	168	38,467	267.57	593	42,746	62.94	724	54,461	28.70	800

**Table 4:** Performance evaluation in a complex environment.

		**Area-Based MCPP Method (ACO)**			**Area-Based MCPP Method (GA)**			**Path-Based MCPP Method (ACO-GA)**		
***m***	***N***	$L_{\mathrm{sum}}\left(\mathrm{pixel}\right)$	$T_{\mathrm{avg}}\left(s\right)$	$T_{\mathrm{idle}}\left(s\right)$	$L_{\mathrm{sum}}\left(\mathrm{pixel}\right)$	$T_{\mathrm{avg}}\left(s\right)$	$T_{\mathrm{idle}}\left(s\right)$	$L_{\mathrm{sum}}\left(\mathrm{pixel}\right)$	$T_{\mathrm{avg}}\left(s\right)$	$T_{\mathrm{idle}}\left(s\right)$
3	70	20,824	15.67	18,761	52,693	8.32	27,421	19,924	31.17	1810
	113	16,755	30.31	6083	67,629	14.42	5307	15,668	86.53	1754
	173	20,684	72.18	5069	72,937	22.12	7991	18,538	231.08	2324
6	70	20,651	8.56	6289	31,050	6.02	10,145	18,856	30.93	1990
	113	17,148	15.81	9663	65,459	11.51	12,576	15,196	86.51	2271
	173	18,215	40.15	10,008	78,579	21.37	13,261	20,095	212.31	2763
10	70	9701	3.67	12,743	11,682	3.04	12,749	12,085	28.68	563
	113	11,791	10.90	16,405	12,632	7.07	16,094	10,116	77.92	861
	173	16,331	29.86	17,050	15,053	13.70	16,918	11,232	208.73	913
		**Path-Based MCPP Method (ACO-ACO)**			**Path-Based MCPP Method (GA-ACO)**			**Path-Based MCPP Method (GA-GA)**		
***m***	***N***	$L_{\mathrm{sum}}\left(\mathrm{pixel}\right)$	$T_{\mathrm{avg}}\left(s\right)$	$T_{\mathrm{idle}}\left(s\right)$	$L_{\mathrm{sum}}\left(\mathrm{pixel}\right)$	$T_{\mathrm{avg}}\left(s\right)$	$T_{\mathrm{idle}}\left(s\right)$	$L_{\mathrm{sum}}\left(\mathrm{pixel}\right)$	$T_{\mathrm{avg}}\left(s\right)$	$T_{\mathrm{idle}}\left(s\right)$
3	70	20,648	62.25	2546	28,999	22.07	1814	45,226	12.07	1729
	113	18,812	112.91	1355	18,979	40.42	895	42,341	25.23	840
	173	19,813	252.24	1863	23,940	135.10	2193	54,858	45.40	1259
6	70	19,221	58.32	2507	24,391	16.92	1949	37,977	11.96	1046
	113	17,691	109.31	1614	18,199	26.86	2375	34,164	24.55	1671
	173	20,346	241.39	1308	18,526	101.48	1839	45,479	44.66	1727
10	70	12,167	31.46	572	16,878	15.52	687	15,550	12.02	1834
	113	10,054	84.75	884	15,378	30.75	1052	15,521	14.67	1247
	173	11,250	243.20	892	18,783	64.16	1609	24,457	22.06	1715

## Data Availability

No applicable.

## Abbreviations

The following abbreviations are used in this manuscript:

MDPI	Multidisciplinary Digital Publishing Institute
DOAJ	Directory of open access journals
TLA	Three letter acronym
LD	Linear dichroism
